# Supplementary material for: Pervasive genetic interactions modulate neurodevelopmental defects of the autism-associated 16p11.2 deletion in Drosophila melanogaster
Source: Nat Commun. 2018 Jun 29;9:2548. doi: 10.1038/s41467-018-04882-6 (PMC6026208; doi:10.1038/s41467-018-04882-6)
Supplement: Supplementary file 1 — Supplementary Information [file 41467_2018_4882_MOESM1_ESM.pdf]

Pervasive genetic interactions modulate neurodevelopmental defects of the autism-associated 16p11.2 deletion in *Drosophila melanogaster*

Iyer *et al*.

Supplementary information

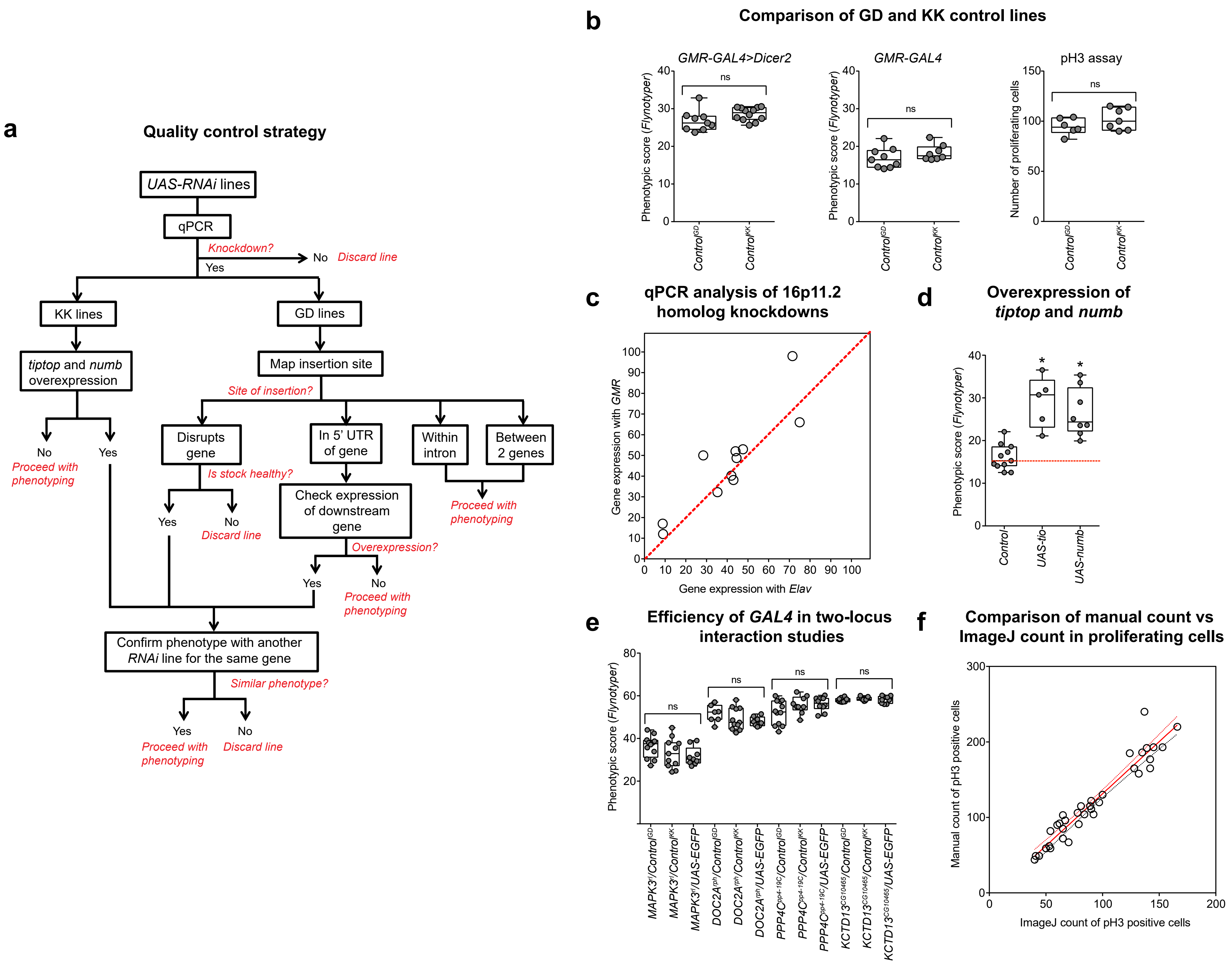
a
Quality control strategy
UAS-RNAi lines
qPCR
Knockdown?
No
Discard line
Yes
KK lines
GD lines
tiptop and numb overexpression
Map insertion site
Site of insertion?
Disrupts gene
In 5' UTR of gene
Within intron
Between 2 genes
No
Proceed with phenotyping
Yes
Is stock healthy?
Yes
No
Discard line
Check expression of downstream gene
Proceed with phenotyping
Overexpression?
Yes
No
Proceed with phenotyping
Confirm phenotype with another RNAi line for the same gene
Similar phenotype?
Yes
Proceed with phenotyping
No
Discard line
b
Comparison of GD and KK control lines
GMR-GAL4>Dicer2
GMR-GAL4
pH3 assay
ns
Phenotypic score (Flynotyper)
Number of proliferating cells
Control^{GD}
Control^{KK}
c
qPCR analysis of 16p11.2 homolog knockdowns
Gene expression with GMR
Gene expression with Elav
d
Overexpression of tiptop and numb
Phenotypic score (Flynotyper)
Control
UAS-tio
UAS-numb
e
Efficiency of GAL4 in two-locus interaction studies
Phenotypic score (Flynotyper)
MAPK3^{rl}/Control^{GD}
MAPK3^{rl}/Control^{KK}
MAPK3^{rl}/UAS-EGFP
DOC2A^{ph}/Control^{GD}
DOC2A^{ph}/Control^{KK}
DOC2A^{ph}/UAS-EGFP
PPP4C$^{pp4-19C}$/Control^{GD}
PPP4C$^{pp4-19C}$/Control^{KK}
PPP4C$^{pp4-19C}$/UAS-EGFP
KCTD13^{CG10465}/Control^{GD}
KCTD13^{CG10465}/Control^{KK}
KCTD13^{CG10465}/UAS-EGFP
f
Comparison of manual count vs ImageJ count in proliferating cells
Manual count of pH3 positive cells
ImageJ count of pH3 positive cells

Supplementary Fig. 1. Quality control of fly RNAi knockdown lines. (a) Quality control strategy for RNAi lines of 16p11.2 homologs. The level of knockdown was checked using qPCR (Supplementary Fig. 1c), followed by insertion site mapping of GD lines and checking *tiptop* and *numb* overexpression in KK lines. We screened a total of 44 RNAi lines for 16p11.2 homologs and selected 31 RNAi lines for our knockdown experiments. **(b)** *Flynotyper* scores of KK and GD control eyes with *GMR-GAL4>Dicer2* and *GMR-GAL4* without *Dicer2,* and number of proliferating cells. We found no significant differences in the phenotypes of GD and KK controls (*n*=7-12, Mann-Whitney test). **(c)** Scatter plot comparing gene expression (mRNA) of 11 homologs of 16p11.2 genes with *GMR-GAL4* and *Elav-GAL4* drivers. A high correlation was observed between knockdown lines using the two drivers (Pearson correlation, r=0.8877). **(d)** Phenotypic scores of *UAS-tio* and *UAS-numb* with *GMR-GAL4* at 30 °C. The *Flynotyper* scores for each overexpression line were significantly higher compared to control (*n*=5-8, Mann-Whitney test, *$p<0.05$). **(e)** *Flynotyper* scores of *UAS-GFP* crosses with 16p11.2 homologs used in two-locus interaction studies show no difference in the eye phenotypes of flies with one *UAS* versus two *UAS* lines (*n*=8-11, Mann-Whitney test). **(f)** Plot comparing manual count versus ImageJ Analyze Particles count of number of proliferating cells. While high correlation (Pearson correlation, r=0.9599, $p<0.0001$) was observed between the two methods, all results shown in the text for number of proliferating cells were counted manually. All boxplots indicate median (center line), 25th and 75th percentiles (bounds of box), and minimum and maximum (whiskers).

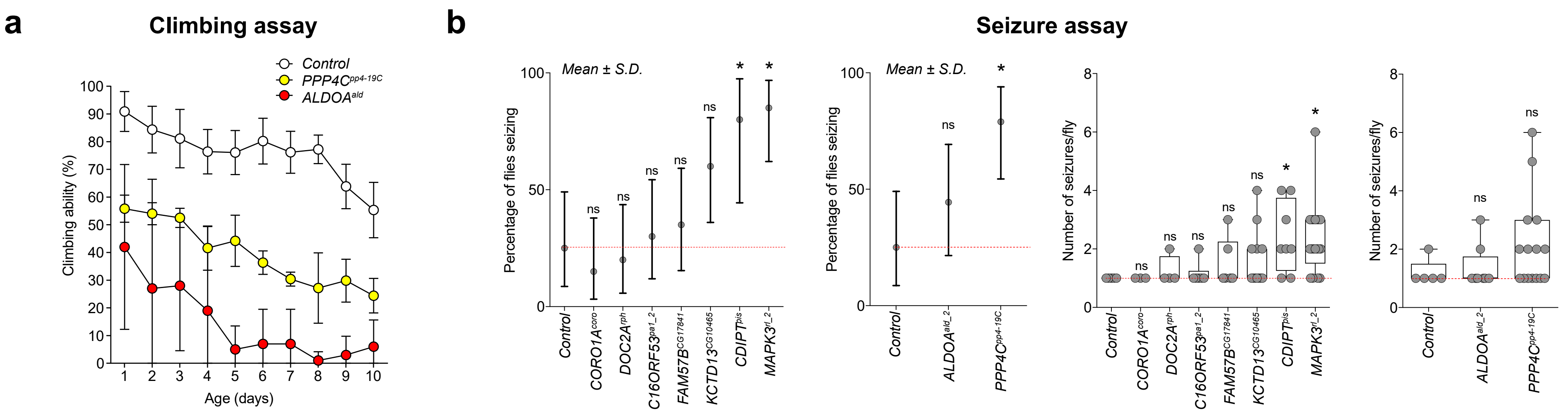

c

NMJ architecture

Gene	NMJ length	NMJ area	NMJ perimeter	Bouton number	Number of branches
FAM57B^{CG17841}	ns	+	ns	+	ns
CDIPT^{pis}	+	ns	ns	+	ns
ALDOA^{ald}	+	ns	ns	ns	+
KCTD13^{CG10465}	ns	ns	ns	ns	ns
C16ORF53^{pa1}	ns	ns	ns	ns	ns
CORO1A^{coro}	ns	ns	ns	ns	ns
DOC2A^{rph}	ns	ns	ns	ns	ns
MAPK3$^{rl\_2}$	ns	ns	ns	ns	ns
PPP4C$^{pp4\text{-}19C}$	ns	ns	ns	ns	ns

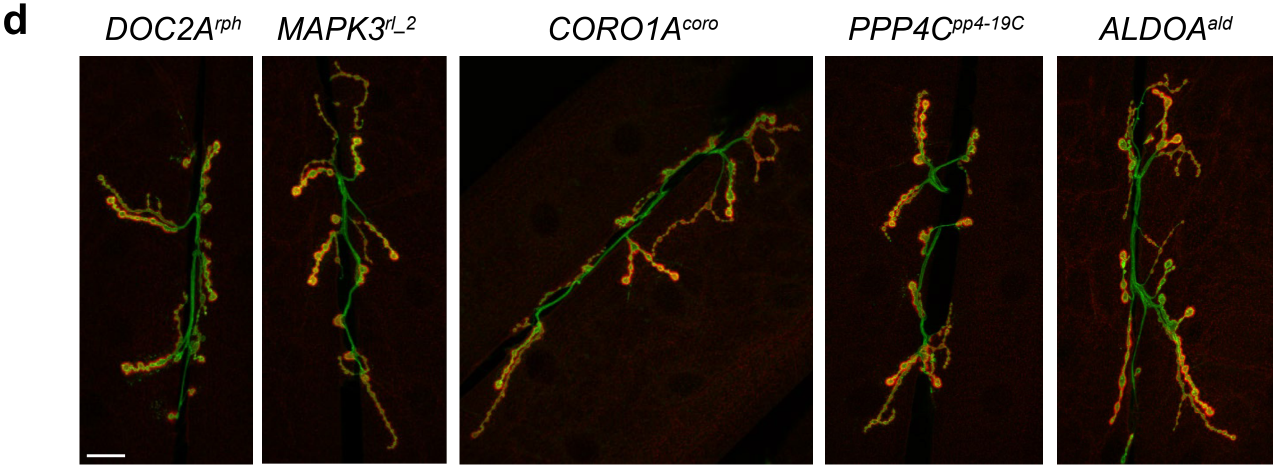

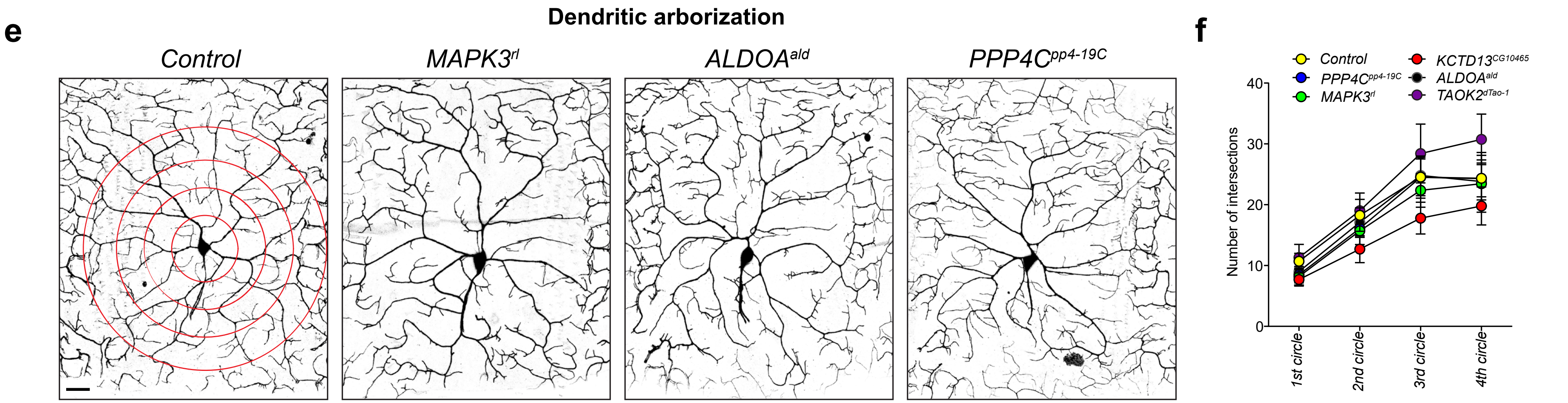

Supplementary Fig. 2. Neurodevelopmental defects in flies with knockdown of individual 16p11.2 homologs. (a) Assessment of motor defects in 16p11.2 homologs with *Elav-GAL4* and no *Dicer2* showed changes in climbing ability over ten days compared to control (two-way ANOVA, F=1.6199, df=27, p=0.0286). Data represented here shows mean ± standard deviation of 10 independent groups of 10 flies for each line. **(b)** Assessment of frequency of spontaneous unprovoked seizure events (Fisher's Exact test, n=10-20, *p<0.05) and average number of seizure events per fly (Mann-Whitney test, n=3-17, *p<0.05) for seven 16p11.2 homologs with *Elav-GAL4>Dicer2* and two homologs, $PPP4C^{pp4\text{-}19C}$ and $ALDOA^{ald}$, with *Elav-GAL4* and no *Dicer2*. Error bars for seizure frequency indicate binomial proportion confidence intervals. Three top homologs were selected for further validation with a larger sample size (Fig. 2c). **(c)** Assessment of neuromuscular junction features for 16p11.2 homologs. "+" indicates a significant change compared to control (p<0.05, Mann-Whitney test), while "ns" indicates no change in that feature. **(d)** Representative confocal images of NMJs for knockdown of five 16p11.2 homologs (scale bar=20 µm). $DOC2A^{rph}$, $MAPK3^{rl}$, and $CORO1A^{coro}$ used *Elav-GAL4>Dicer2* at 25°C, while $PPP4C^{pp4\text{-}19C}$ and $ALDOA^{ald}$ used *Elav-GAL4* without *Dicer2* at room temperature. **(e)** Representative confocal live images of class IV da neurons labeled with *mCD8-GFP* under the control of *ppk-GAL4* are shown for three 16p11.2 homologs and control (scale bar=25 µm). Red circles on the control image indicate the circles used in Sholl analysis. **(f)** Line plot of the number of intersections at each concentric circle (two-way ANOVA, p=0.0027, df=5, F=3.76). Intersections were calculated by manual Sholl analysis. Data represented here shows mean ± standard deviation of 10-16 flies per genotype for each circle. All boxplots indicate median (center line), 25th and 75th percentiles (bounds of box), and minimum and maximum (whiskers).

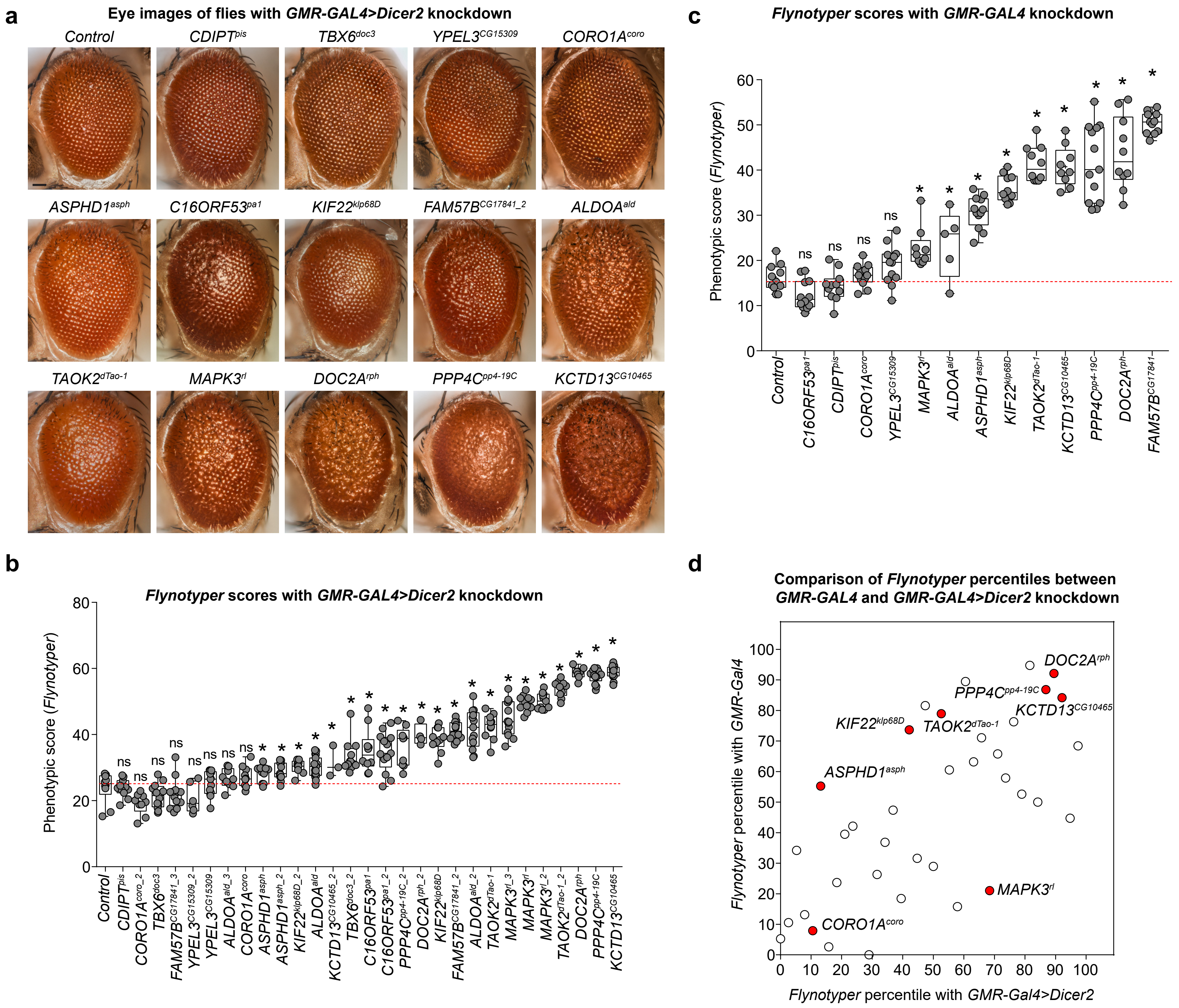
a
Eye images of flies with GMR-GAL4>Dicer2 knockdown
Control
CDIPTpis
TBX6doc3
YPEL3CG15309
CORO1Acoro
ASPHD1asph
C16ORF53pa1
KIF22klp68D
FAM57BCG17841_2
ALDOAald
TAOK2dTao-1
MAPK3rl
DOC2Arph
PPP4Cpp4-19C
KCTD13CG10465
b
Flynotyper scores with GMR-GAL4>Dicer2 knockdown
Phenotypic score (Flynotyper)
Control
CDIPTpis
CORO1Acoro_2
TBX6doc3
FAM57BCG17841_3
YPEL3CG15309_2
YPEL3CG15309
ALDOAald_3
CORO1Acoro
ASPHD1asph
ASPHD1asph_2
KIF22klp68D_2
ALDOAald
KCTD13CG10465_2
TBX6doc3_2
C16ORF53pa1
C16ORF53pa1_2
PPP4Cpp4-19C_2
DOC2Arph_2
KIF22klp68D
FAM57BCG17841_2
ALDOAald_2
TAOK2dTao-1
MAPK3rl_3
MAPK3rl
MAPK3rl_2
TAOK2dTao-1_2
DOC2Arph
PPP4Cpp4-19C
KCTD13CG10465
c
Flynotyper scores with GMR-GAL4 knockdown
Phenotypic score (Flynotyper)
Control
C16ORF53pa1
CDIPTpis
CORO1Acoro
YPEL3CG15309
MAPK3rl
ALDOAald
ASPHD1asph
KIF22klp68D
TAOK2dTao-1
KCTD13CG10465
PPP4Cpp4-19C
DOC2Arph
FAM57BCG17841
d
Comparison of Flynotyper percentiles between GMR-GAL4 and GMR-GAL4>Dicer2 knockdown
Flynotyper percentile with GMR-Gal4
Flynotyper percentile with GMR-Gal4>Dicer2
DOC2Arph
PPP4Cpp4-19C
KCTD13CG10465
KIF22klp68D
TAOK2dTao-1
ASPHD1asph
MAPK3rl
CORO1Acoro

Supplementary Fig. 3. Adult eye phenotypes of one-hit knockdown models of 16p11.2 homologs. (a) Representative brightfield microscope images of fly eyes displaying eye-specific knockdown for each 16p11.2 homolog at 30°C with *GMR-GAL4>Dicer2* driver (scale bar=50 µm). **(b)** *Flynotyper* scores for 29 tested *GMR-GAL4>Dicer2* lines of 16p11.2 homologs. Twenty-one lines had significantly higher *Flynotyper* scores compared to the control (n=3-19, Mann-Whitney test, *p<0.05). Two tested lines, $FAM57B^{CG17841}$ (lethal) and $CDIPT^{pis\_2}$, are not shown in this figure. **(c)** Box plot of *Flynotyper* scores for knockdown of 13 tested homologs of 16p11.2 genes with *GMR-GAL4* and no *Dicer2*. Nine of the 13 tested homologs had significantly higher *Flynotyper* scores than the control (n=5-13, Mann-Whitney test, *p<0.05). **(d)** Comparison of percentile ranks for *Flynotyper* scores of *GMR-GAL4* lines of 16p11.2 homologs and other neurodevelopmental genes with and without *Dicer2* (Supplementary Table 2). Select 16p11.2 genes are highlighted in red. Percentiles of homologs with *Dicer2* correlate to percentiles of homologs without *Dicer2* (Pearson correlation, r=0.6853, $p=2.877\times10^{-6}$), showing that relative severity of the eye phenotype is not affected by *Dicer2*. All boxplots indicate median (center line), 25th and 75th percentiles (bounds of box), and minimum and maximum (whiskers).

Cellular defects in flies with one-hit knockdown of 16p11.2 homologs

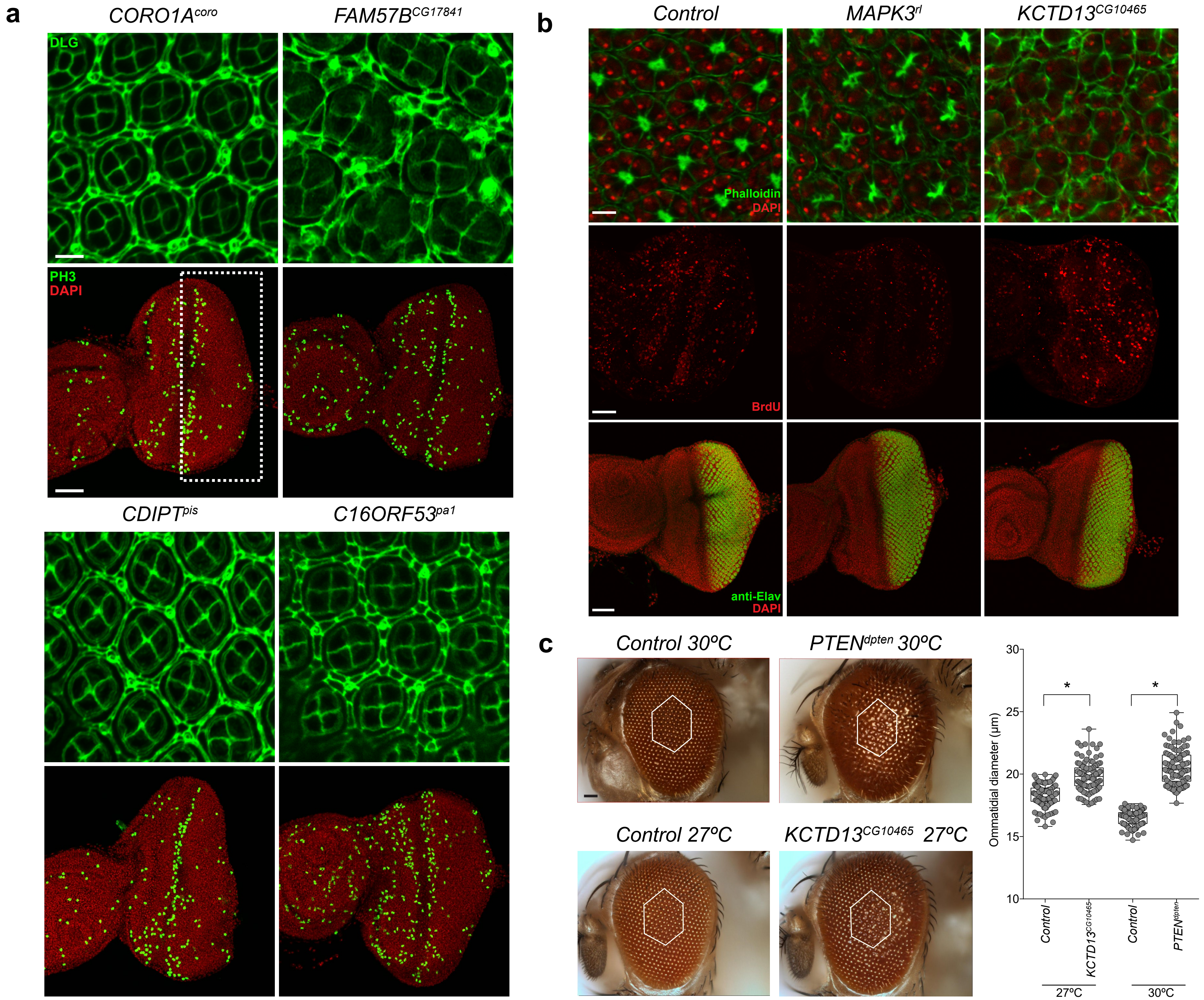

Supplementary Fig. 4. Cellular eye phenotypes of one-hit knockdown models of 16p11.2 homologs. (a) Representative confocal images of the pupal eye (scale bar=5 µm) and larval eye disc (scale bar=30 µm) illustrate the defects observed in eye-specific knockdown of four 16p11.2 homologs with *GMR-GAL4* driver and no *Dicer2* (other homologs are shown in Figs. 5a and 6e). **(b)** Representative confocal images of pupal eyes stained with phalloidin and DAPI (scale bar=5 µm) for 16p11.2 homologs illustrate defects in photoreceptor cell counts, and larval eye discs stained with BrdU and anti-Elav (scale bar=30 µm) show the effects of knockdown on cell proliferation and differentiation, respectively. No significant differences in the number of differentiating cells were observed in either knockdown when compared to control. **(c)** Eye images of control, $PTEN^{dpten}$ and $KCTD13^{CG10465}$ knockdown flies (scale bar=50 µm) highlighting regions (white hexagons) used to calculate the average size of each ommatidium. $KCTD13^{CG10465}$ knockdown flies were raised at 27 °C due to severity of the eye phenotype at 30 °C. Both knockdowns showed an increase in ommatidial diameter compared to their respective controls (n=100, *p<0.05, Mann-Whitney test). Boxplot indicates median (center line), 25th and 75th percentiles (bounds of box), and minimum and maximum (whiskers).

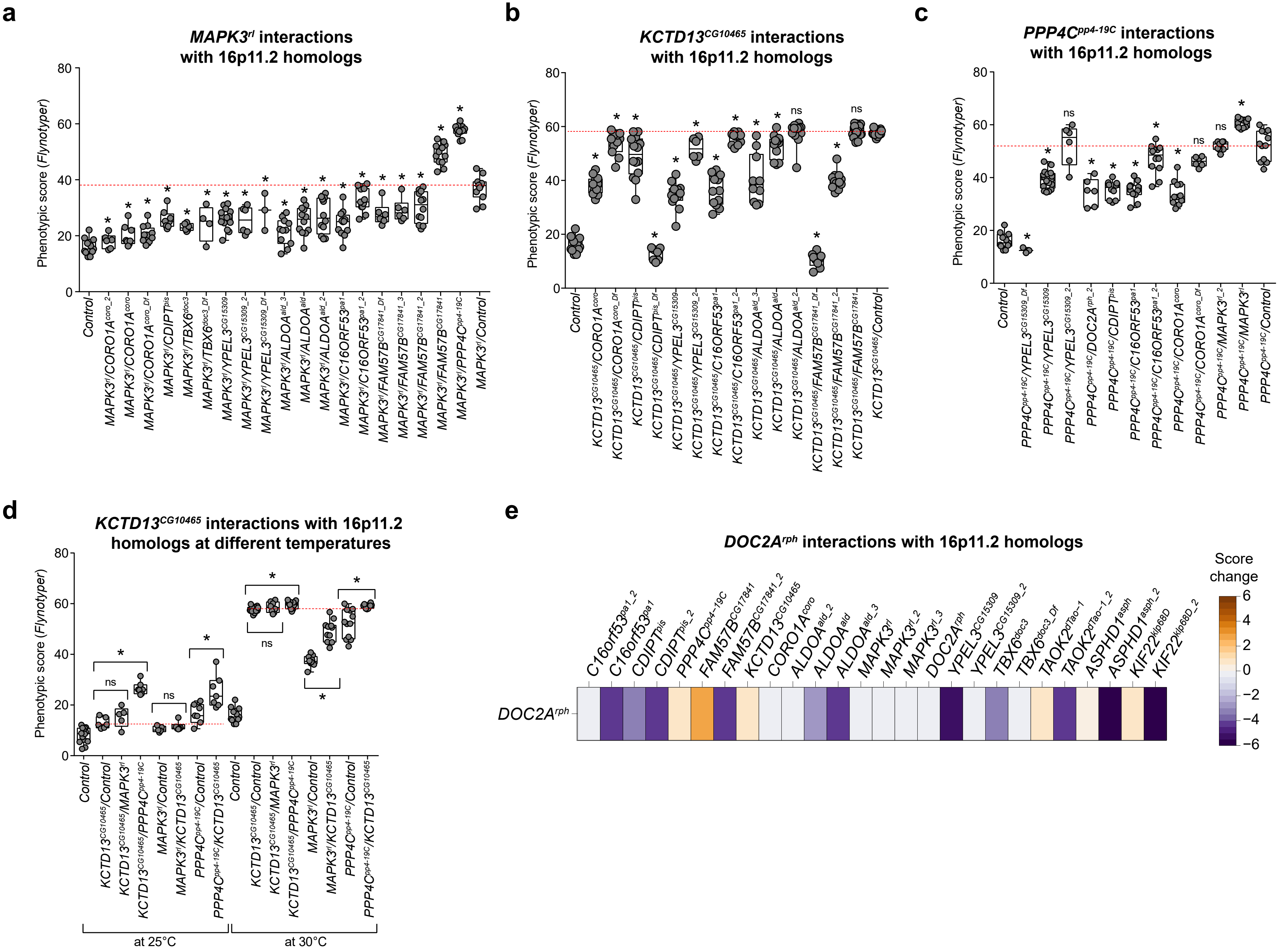
a
MAPK3rl interactions with 16p11.2 homologs
Phenotypic score (Flynotyper)
Control
MAPK3rl/CORO1Acoro_2
MAPK3rl/CORO1Acoro
MAPK3rl/CORO1Acoro_Df
MAPK3rl/CDIPTpis
MAPK3rl/TBX6doc3
MAPK3rl/TBX6doc3_Df
MAPK3rl/YPEL3CG15309
MAPK3rl/YPEL3CG15309_2
MAPK3rl/YPEL3CG15309_Df
MAPK3rl/ALDOAald_3
MAPK3rl/ALDOAald
MAPK3rl/ALDOAald_2
MAPK3rl/C16ORF53pa1
MAPK3rl/C16ORF53pa1_2
MAPK3rl/FAM57BCG17841_Df
MAPK3rl/FAM57BCG17841_3
MAPK3rl/FAM57BCG17841_2
MAPK3rl/FAM57BCG17841
MAPK3rl/PPP4Cpp4-19C
MAPK3rl/Control
b
KCTD13CG10465 interactions with 16p11.2 homologs
Phenotypic score (Flynotyper)
Control
KCTD13CG10465/CORO1Acoro
KCTD13CG10465/CORO1Acoro_Df
KCTD13CG10465/CDIPTpis
KCTD13CG10465/CDIPTpis_Df
KCTD13CG10465/YPEL3CG15309
KCTD13CG10465/YPEL3CG15309_2
KCTD13CG10465/C16ORF53pa1
KCTD13CG10465/C16ORF53pa1_2
KCTD13CG10465/ALDOAald_3
KCTD13CG10465/ALDOAald
KCTD13CG10465/ALDOAald_2
KCTD13CG10465/FAM57BCG17841_Df
KCTD13CG10465/FAM57BCG17841_2
KCTD13CG10465/FAM57BCG17841
KCTD13CG10465/Control
c
PPP4Cpp4-19C interactions with 16p11.2 homologs
Phenotypic score (Flynotyper)
Control
PPP4Cpp4-19C/YPEL3CG15309_Df
PPP4Cpp4-19C/YPEL3CG15309
PPP4Cpp4-19C/YPEL3CG15309_2
PPP4Cpp4-19C/DOC2Arph_2
PPP4Cpp4-19C/CDIPTpis
PPP4Cpp4-19C/C16ORF53pa1
PPP4Cpp4-19C/C16ORF53pa1_2
PPP4Cpp4-19C/CORO1Acoro
PPP4Cpp4-19C/CORO1Acoro_Df
PPP4Cpp4-19C/MAPK3rl_2
PPP4Cpp4-19C/MAPK3rl
PPP4Cpp4-19C/Control
d
KCTD13CG10465 interactions with 16p11.2 homologs at different temperatures
Phenotypic score (Flynotyper)
Control
KCTD13CG10465/Control
KCTD13CG10465/MAPK3rl
KCTD13CG10465/PPP4Cpp4-19C
MAPK3rl/Control
MAPK3rl/KCTD13CG10465
PPP4Cpp4-19C/Control
PPP4Cpp4-19C/KCTD13CG10465
at 25°C
at 30°C
e
DOC2Arph interactions with 16p11.2 homologs
C16orf53pa1_2
C16orf53pa1
CDIPTpis
CDIPTpis_2
PPP4Cpp4-19C
FAM57BCG17841
FAM57BCG17841_2
KCTD13CG10465
CORO1Acoro
ALDOAald_2
ALDOAald
ALDOAald_3
MAPK3rl
MAPK3rl_2
MAPK3rl_3
DOC2Arph
YPEL3CG15309
YPEL3CG15309_2
TBX6doc3
TBX6doc3_Df
TAOK2dTao-1
TAOK2dTao-1_2
ASPHD1asph
ASPHD1asph_2
KIF22klp68D
KIF22klp68D_2
DOC2Arph
Score change

Supplementary Fig. 5. *Flynotyper* screening of interactions between 16p11.2 homologs. (a) Representative *Flynotyper* scores for 19 crosses of $MAPK3^{rl}$ with eight 16p11.2 homologs that modify the one-hit $MAPK3^{rl}$ phenotype (n=3-15, Mann-Whitney test, *p<0.05). Concordant results from two or more lines were required to identify positive interactions. Because single knockdown of $FAM57B^{CG17841}$ is partially lethal, the two-hit model with $MAPK3^{rl}$ is considered suppression. **(b)** Representative *Flynotyper* scores for 14 crosses of $KCTD13^{CG10465}$ with six 16p11.2 homologs that modify the one-hit $KCTD13^{CG10465}$ phenotype (n=3-17, Mann-Whitney test, *p<0.05). Because RNAi knockdown of $FAM57B^{CG17841}$ is partially lethal, the two-hit model with $KCTD13^{CG10465}$ is considered as a suppression of the lethality phenotype. **(c)** Representative *Flynotyper* scores for 11 crosses of $PPP4C^{pp4\text{-}19C}$ with six 16p11.2 homologs that modify the one-hit $PPP4C^{pp4\text{-}19C}$ phenotype (n=6-20, Mann-Whitney test, *p<0.05). Even though only one line each of $MAPK3^{rl}$ and $CORO1A^{coro}$ showed significant changes compared to the one-hit control, the second lines tested showed suppression of the glossy eye phenotype or a trend towards enhancement of the phenotype, respectively, which were indicative of validated interactions. **(d)** Representative *Flynotyper* scores for $KCTD13^{CG10465}$ two-hit crosses with $MAPK3^{rl}$ and $PPP4C^{pp4\text{-}19C}$ at different temperatures (n=5-13, Mann-Whitney test, *p<0.05). We suspected that the enhanced eye phenotypes at 30 °C were due to severity of $KCTD13^{CG10465}$ knockdown, and confirmed a significant enhancement of the eye phenotype for $KCTD13^{CG10465/}PPP4C^{pp4\text{-}19C}$ at 25 °C. **(e)** A heat map of the change in manual eye scores of $DOC2A^{rph}$ two-hit knockdowns with other 16p11.2 homologs compared to $DOC2A^{rph}$ one-hit flies.

Cellular phenotypes of 16p11.2 two-hit models

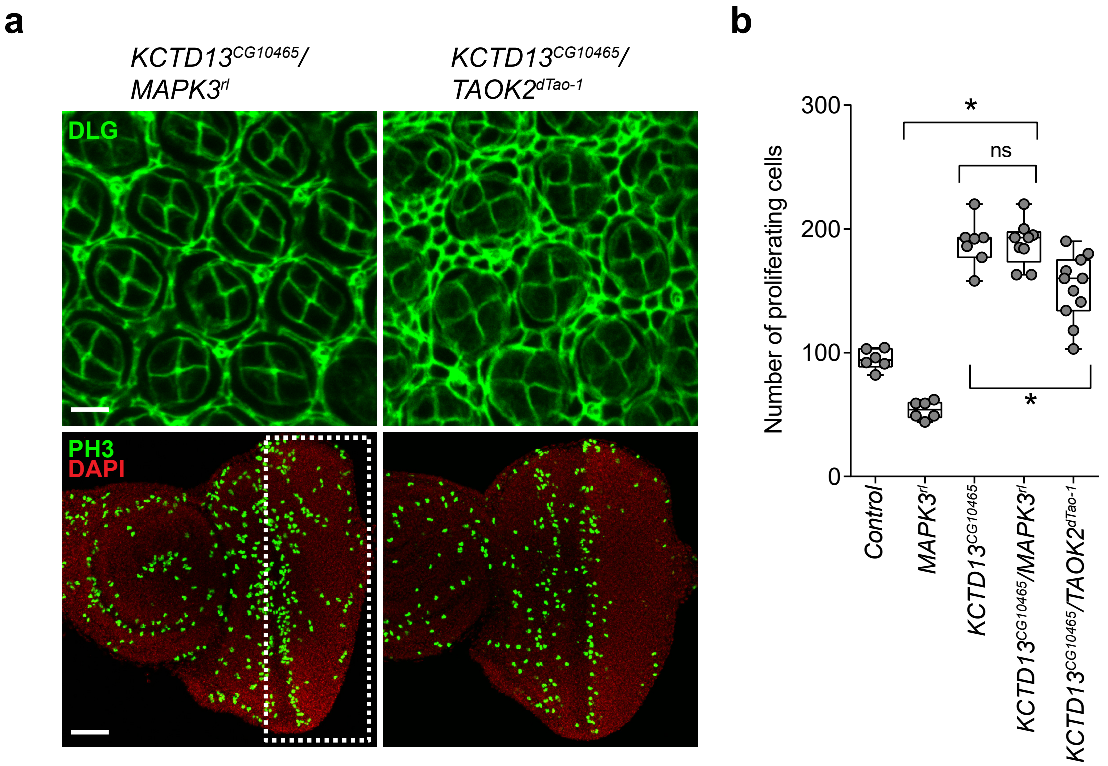

c

Gene	Cone cell defects	Primary cell defects	Bristle group defects	Secondary cell defects	Tertiary cell defects	Rotation defects	Hexagon structure defects
MAPK3^{rl}		+	+	++	+	+	+
MAPK3^{rl}/ *ALDOA*^{ald}		Supp	Supp	+	Supp	Supp	Supp
MAPK3^{rl}/ *TAOK2*$^{dTao-1}$		Supp	+	+	+	+	+
KCTD13^{CG10465}	+++	+	++	++	+	+	++
KCTD13^{CG10465}/ *ALDOA*^{ald}	Supp	Supp	++	Supp	Supp	Supp	Supp
KCTD13^{CG10465}/ *TAOK2*$^{dTao-1}$	Supp	Supp	++	++	+	+	++
KCTD13^{CG10465}/ *MAPK3*^{rl}	Supp	Supp	++	++	++	+	++

Human genetic interaction networks of 16p11.2 genes

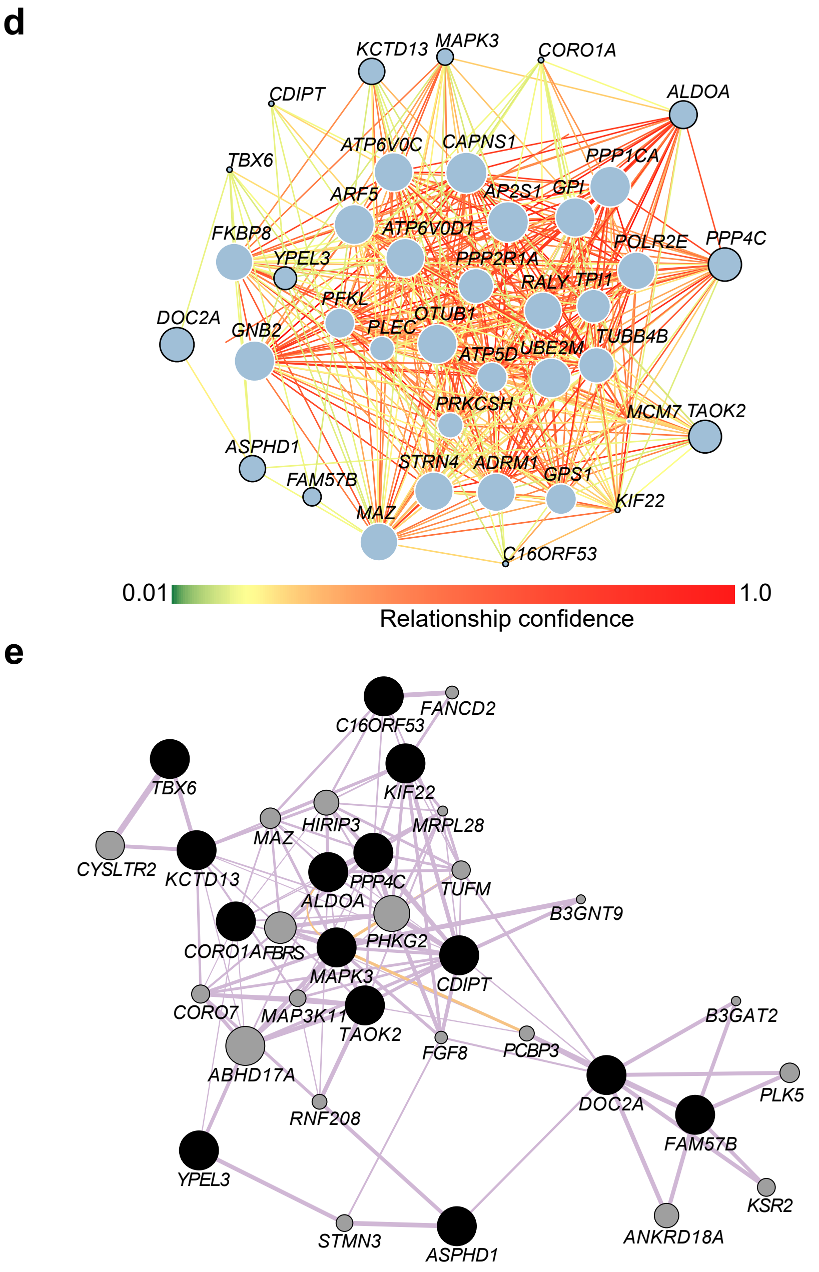

Supplementary Fig. 6. Cellular phenotypes and human gene interactions of 16p11.2 homolog pairs. (a) Representative confocal images of pupal eye (scale bar=5 µm) and larval eye disc (scale bar=30 µm), stained with anti-Dlg and anti-pH3 respectively, of double knockdown of $KCTD13^{CG10465}/MAPK3^{rl}$ and $KCTD13^{CG10465}/TAOK2^{dTao\text{-}1}$. **(b)** Assessment of proliferating cell count in the larval eyes shown in Supplementary Fig. 6A (n=9-11, Mann-Whitney test, $*p<0.05$). Boxplot indicates median (center line), 25th and 75th percentiles (bounds of box), and minimum and maximum (whiskers). **(c)** Table summarizing the cellular defects observed in the pupal eyes of one-hit and two-hit 16p11.2 homologs knockdown. "+" symbols indicate the severity of the observed cellular defects, while "Supp" indicates that the cellular defects were suppressed in the two-hit models. **(d)** Interaction network of fourteen tested 16p11.2 genes in the context of a brain-specific gene interaction network[1]. Nodes with black borders represent 16p11.2 genes, while white borders represent 25 connector genes. The size of the nodes represents a predictive score for the involvement of each gene in autism, and colors of the edges represent confidence in the annotated interactions. **(e)** Interaction network of fourteen tested 16p11.2 genes in the context of a general human gene interaction network (GeneMania)[2]. Black-shaded nodes represent the input 16p11.2 genes, while grey nodes represent the connector genes. Edge color represents interaction data source (purple: co-expression, orange: predicted interaction). Note that the 16p11.2 distal gene *TUFM* is connected to *MAPK3*, *C16ORF53*, and *DOC2A*, consistent with the interactions identified in fly models.

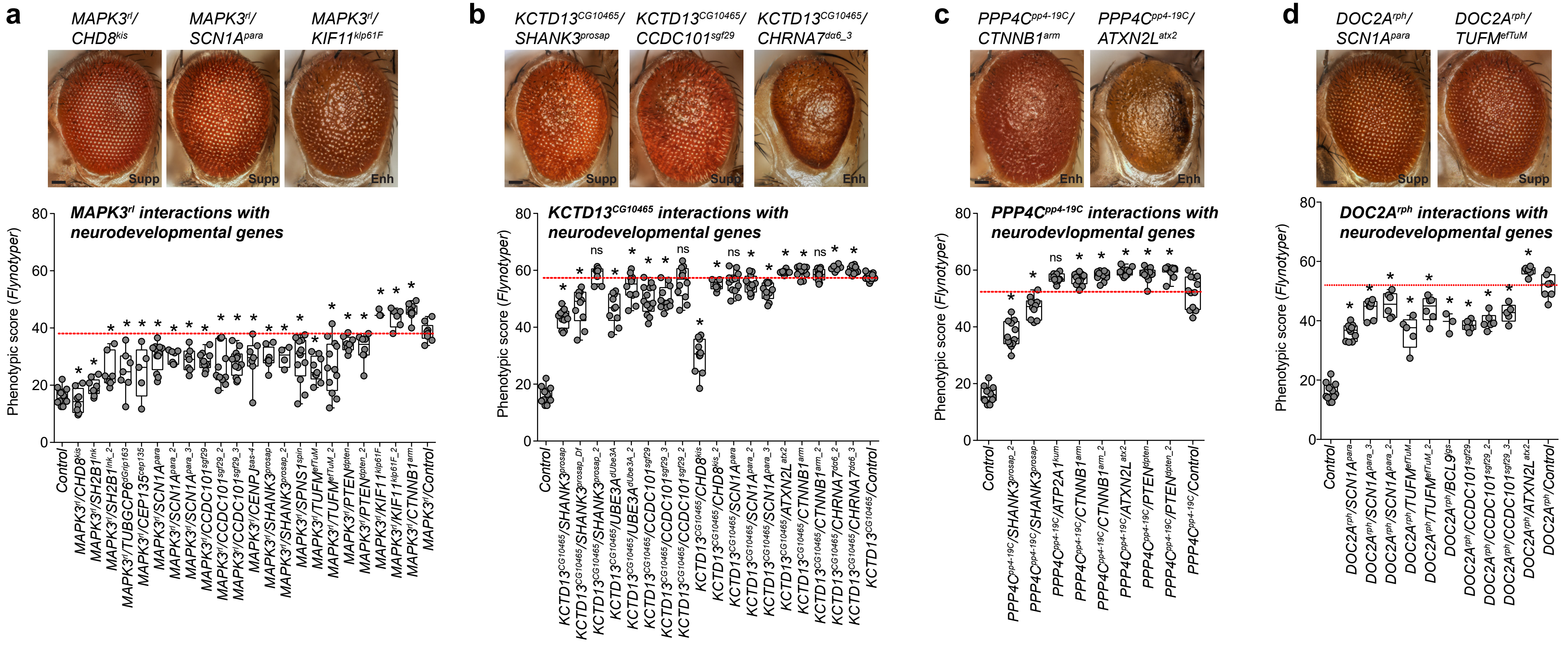

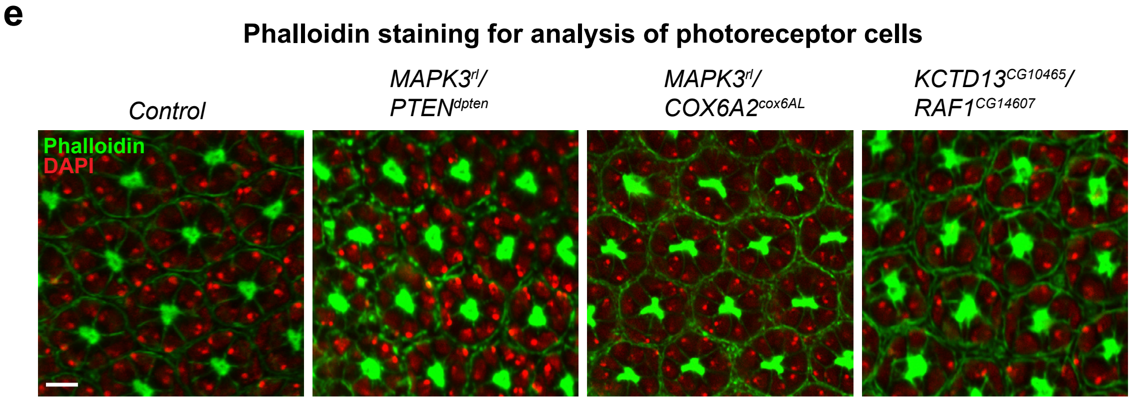

f

Cellular defects with two-hit knockdown of 16p11.2 homologs and neurodevelopmental genes

**Gene**	Cone cell defects	Primary cell defects	Bristle group defects	Secondary cell defects	Tertiary cell defects	Rotation defects	Hexagon structure defects
$MAPK3^{rl}$		+	+	++	+	+	+
$MAPK3^{rl}$/ $PTEN^{dpten}$		Supp	+	Supp	Supp	Supp	Supp
$MAPK3^{rl}$/ $COX6A2^{cox6AL}$		Supp	+	Supp	Supp	Supp	Supp
$KCTD13^{CG10465}$	+++	+	++	++	+	+	++
$KCTD13^{CG10465}$/ $RAF1^{CG14607}$	Supp	Supp	++	++	+	Supp	+

Supplementary Fig. 7. Interactions of 16p11.2 homologs with neurodevelopmental genes. (a) Representative eye images (scale bar=50 µm) and *Flynotyper* scores for 22 crosses of $MAPK3^{rl}$ with 13 neurodevelopmental genes that modify the one-hit $MAPK3^{rl}$ phenotype (*n*=3-12, Mann-Whitney test, *$p<0.05$). Concordant results from two or more lines were required to identify positive interactions. **(b)** Representative eye images (scale bar=50 µm) and *Flynotyper* scores for 18 crosses of $KCTD13^{CG10465}$ with eight neurodevelopmental genes that modify the one-hit $KCTD13^{CG10465}$ phenotype (*n*=6-18, Mann-Whitney test, *$p<0.05$). While the *Flynotyper* score of $KCTD13^{CG10465}/CTNNB1^{arm\_2}$ is not significant, it is considered to be an enhancer since black spots were observed in the eyes with double knockdown. **(c)** Representative eye images (scale bar=50 µm) and *Flynotyper* scores for eight crosses of $PPP4C^{pp4\text{-}19C}$ with five neurodevelopmental genes that modify the one-hit $PPP4C^{pp4\text{-}19C}$ phenotype (*n*=10-12, Mann-Whitney test, *$p<0.05$). While $PPP4C^{pp4\text{-}19C}/ATP2A1^{kum}$ did not have a significant *Flynotyper* score, we considered this interaction to be an enhancement due to its glossy eye phenotype. **(d)** Representative eye images (scale bar=50 µm) and *Flynotyper* scores for 10 crosses of $DOC2A^{rph}$ with five neurodevelopmental genes that modify the one-hit $DOC2A^{rph}$ phenotype (*n*=3-10, Mann-Whitney test, *$p<0.05$). **(e)** Representative confocal images of pupal eyes stained with phalloidin and DAPI in control and double knockdowns of $MAPK3^{rl}/PTEN^{dpten}$, $MAPK3^{rl}/COX6A2^{cox6AL}$ and $KCTD13^{CG10465}/RAF1^{CG14607}$ (scale bar=5 µm). **(f)** Table summarizing the cellular defects observed in the pupal eye of one-hit $MAPK3^{rl}$ and $KCTD13^{CG10465}$ flies compared to double knockdown of $MAPK3^{rl}/PTEN^{dpten}$, $MAPK3^{rl}/COX6A2^{cox6AL}$ and $KCTD13^{CG10465}/RAF1^{CG14607}$. "+" symbols indicate the severity of the observed cellular defects, while "Supp" indicates that the cellular defects were suppressed in the two-hit models. All boxplots indicate median (center line), 25th and 75th percentiles (bounds of box), and minimum and maximum (whiskers).

a

Enriched gene ontology terms in differentially-expressed genes of 16p11.2 homologs

b

Network of cell proliferation genes differentially expressed in 16p11.2 homologs

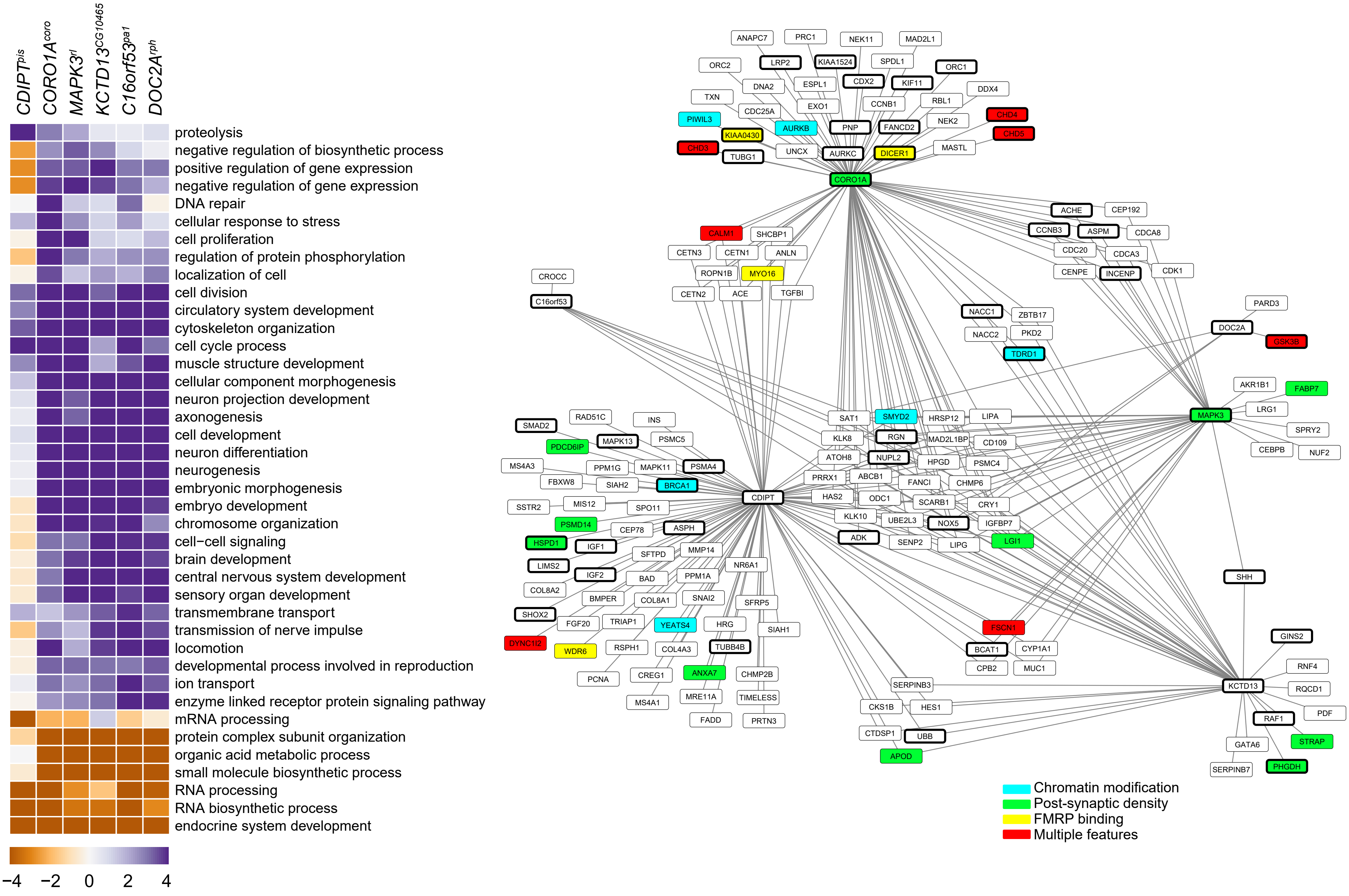

Supplementary Fig. 8. Functional enrichment of differentially-expressed genes in 16p11.2 knockdowns. (a) Heatmap showing selected Gene Ontology biological process terms whose component genes have significantly higher (blue) or lower (orange) expression values in the six 16p11.2 homolog knockdowns compared to wild-type control ($p<0.01$ corrected by Benjamini-Hochberg method, Parametric Analysis of Geneset Enrichment test). **(b)** Network of all differentially-expressed cell proliferation genes in each 16p11.2 knockdown model. Edges connect 16p11.2 genes (hub nodes) to differentially-expressed genes (outer nodes). Thick node borders indicate genes related to neurodevelopmental diseases, while the node color represents neurodevelopmental function of those genes.

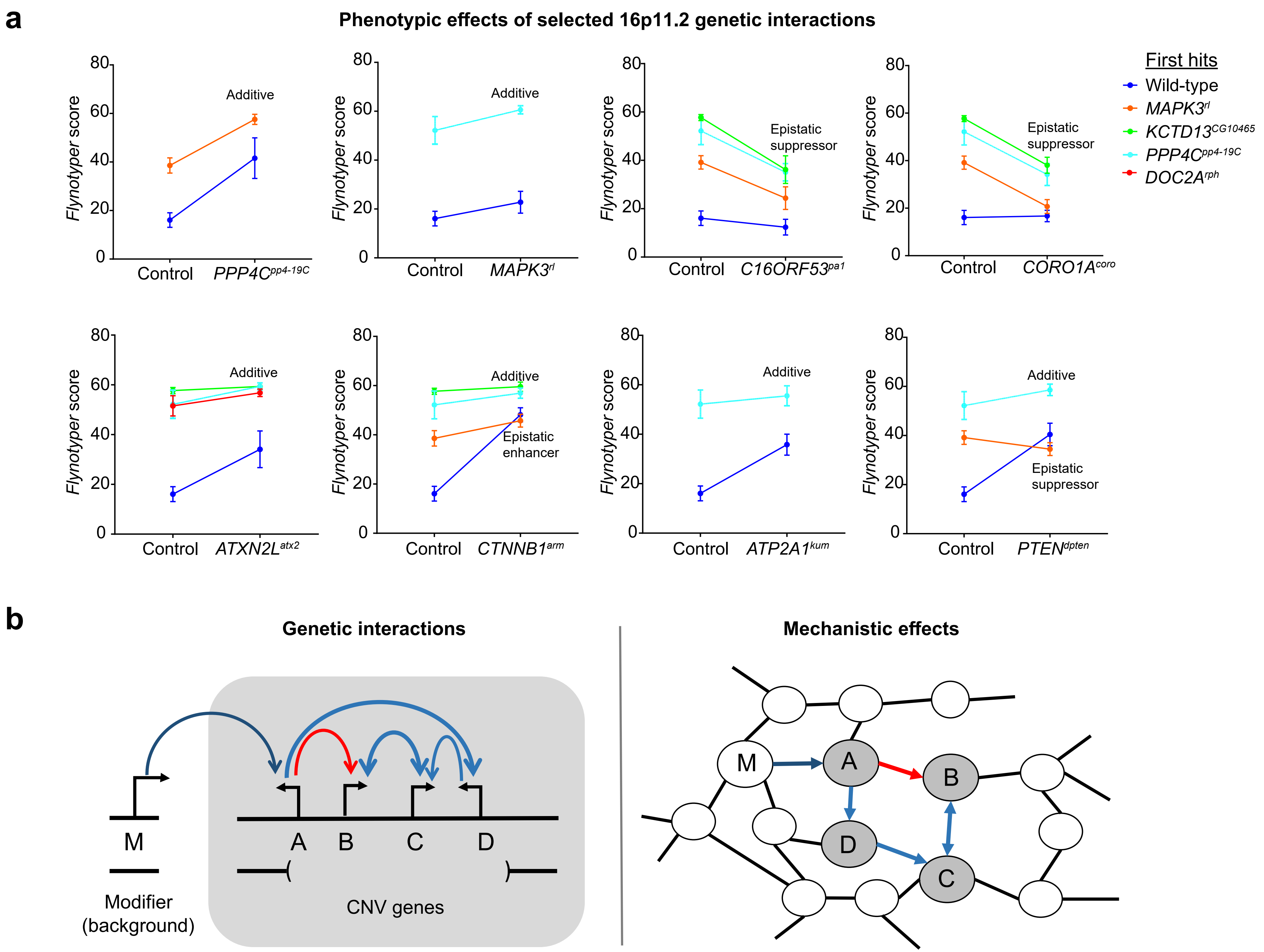
a
Phenotypic effects of selected 16p11.2 genetic interactions
First hits
Wild-type
MAPK3rl
KCTD13CG10465
PPP4Cpp4-19C
DOC2Arph
Flynotyper score
0
20
40
60
80
Control
PPP4Cpp4-19C
MAPK3rl
C16ORF53pa1
CORO1Acoro
ATXN2Latx2
CTNNB1arm
ATP2A1kum
PTENdpten
Additive
Epistatic suppressor
Epistatic enhancer
b
Genetic interactions
Mechanistic effects
M
A
B
C
D
Modifier (background)
CNV genes

Supplementary Fig. 9. A complex interaction model for pathogenicity of 16p11.2 deletion. (a) Representative *Flynotyper* phenotypes of 16p11.2 pairwise knockdowns showing enhancement or suppression of the one-hit phenotypes in the *Drosophila* eye. Error bars indicate mean ± standard deviation of *Flynotyper* scores for the selected genotypes (*n*=6-14 samples). Epistatic enhancer and suppressor interactions were confirmed using two-way ANOVA tests ($p<0.05$, df=1, $F>4.5202$; see Supplementary Data 8). **(b)** In the human 16p11.2 deletion, modifier genes elsewhere in the genome can modulate the effects of multiple CNV genes (left) through shared cellular pathways (right), allowing complex interactions between pairs of CNV genes to ultimately influence the observed neurodevelopmental phenotypes. Colored arrows represent suppression, blue, or enhancement, red, of the phenotypes caused by single-gene knockdown.

SUPPLEMENTARY TABLES

Supplementary Table 1. *Drosophila* homologs and model organism phenotypes of 16p11.2 genes

	Human gene	Fly ortholog	DIOPT score	Query coverage	Percentage identity (aa)	E value	*Drosophila* phenotypes							Zebrafish phenotypes			Mouse phenotypes		
							Early stage lethality (larval/pupal)	Developmental defects	Eye morphology defects	Neuronal defects	Motor defects	Expression in developing eye	Expression in adult eye	Neuroanatomical defects	Motor defects	Brain morphology defects	Developmental defects	Neuronal defects	Other defects *
1	*CDIPT*	*CG9245 (pis)*	10.00	98%	51%	2.00E-71	+	+		+	+	+	+			+			+
2	*PPP4C*	*CG32505 (pp4-19C)*	9.00	100%	92%	0	+	+	++	+	+	+	+		+	+	+ L		+
3	*ALDOA*	*CG6058 (ald)*	9.00	99%	69%	0	+	+	+	+	+	+	+		+	+	ND	ND	ND
4	*YPEL3*	*CG15309*	7.00	94%	82%	1.00E-64		+				+	+		+	+		+	+
5	*FAM57B*	*CG17841*	7.00	93%	30%	2.00E-27		+	++	+	+	+	+	+	+	+	ND	ND	ND
6	*CORO1A*	*CG9446 (coro)*	7.00	100%	50%	2.00E-173	+	+		+	+	+	+		+	+		+	+
7	*INO80E*	*CG18004* [1]	7.00	29%	65%	2.00E-12													
8	*MAPK3*	*CG12559 (rl)*	6.00	89%	82%	0		+	+	+	+	+	+		+	+	+	+	+
9	*KCTD13*	*CG10465*	6.00	79%	63%	1.00E-117		+	++	++	+	+	+	+	+	+	ND	ND	ND
10	*TAOK2*	*CG14217 (dTao-1)*	6.00	31%	68%	7.00E-165	+	+	+	+	ND	+	+			+		+	+
11	*TBX6*	*CG5093 (doc3)*	6.00	43%	58%	5.00E-76	+	+	+		+	+	+		+	+	+	+	+
12	*DOC2A*	*CG11556 (rph)*	5.00	75%	38%	9.00E-64		+	++	+	+		+	+		+		+	
13	*ASPHD1*	*CG8421 (asph)*	3.00	33%	38%	5.00E-19				ND	+		+			+	ND	ND	ND
14	*C16ORF53*	*CG11750 (pa1)*	3.00					+	+	+	+	+	+			+	ND	ND	ND
15	*KIF22*	*CG7293 (klp68D)*	-	53%	41%	2.00E-64	+	+	+	+	ND	+	+		+	+	+		+
16	*MAZ*	*CG14938 (crol)*	1.00	62%	26%	1.00E-29													
17	*SEZ6L2*	*CG1500 (fw)*	-	29%	30%	7.00E-13													
18	*GDPD3*	*CG3942*	-	14%	39%	6.00E-12													

19	*SPN*	*CG13409*	-	31%	28%	1.8													
20	*C16ORF54*	*CG6621*	-	25%	33%	1.5													
21	*TMEM219*	*CG6280*	-	14%	29%	0.010													
22	*QPRT*	*CG15730*	-	8%	41%	0.037													
23	*PRRT2*	*No ortholog*	-																
24	*MVP*	*No ortholog*	-																
25	*HIRIP3*	*No ortholog*	-																

[1] No RNAi lines were available for *CG18004*.
*Cellular, immunological, cardiovascular, mortality/aging, hematopoietic, muscular, hearing/vestibular/ear, limbs/digits/tail, endocrine/exocrine glands, renal/urinary system, homeostasis/metabolism

We queried the *Drosophila* genome for homologs using DRSC Integrative Ortholog Prediction Tool (DIOPT)[3], ENSEMBL database[4] and reciprocal BLAST for each of the 25 human 16p11.2 genes. We selected 15 homologs with a DIOPT score of 3.00 or greater, or in the case of $KIF22^{klp68D}$, both high query coverage and percentage identity in BLAST. Three genes did not have any homologs, and the remaining seven genes with low or no DIOPT scores had low (<30%) query coverage and/or percentage identity in BLAST. Of the 15 selected homologs, one homolog ($INO80E^{CG18004}$) did not have any available RNAi lines. Overall, fly lines of 14 homologs for human 16p11.2 genes were used in this study. Phenotypes due to single gene knockdown or knockout in *Drosophila*, mouse[5, 6] and zebrafish[7, 8, 9] models of the 14 selected homologs are also shown; +: degree of phenotype severity, L: lethality; ND: phenotype not determined. Gene expression in the *Drosophila* eye was determined from FlyAtlas[10] (adult eye) and modEncode expression data[11] (imaginal disc, larvae L3 wandering) or from previous functional studies[12, 13, 14, 15].

Supplementary Table 2. Percentiles of *Flynotyper* scores for one-hit knockdown flies with *Dicer2*

	Gene category	*Flynotyper*	Rank	Percentile
$ATXN2L^{atx2}$	16p11.2 distal	63.39	1	97.37%
$UBE3A^{dUbe3A}$	Core gene	59.05	2	94.74%
$KCTD13^{CG10465}$	16p11.2	58.78	3	92.11%
$DOC2A^{rph}$	16p11.2	58.61	4	89.47%
$PPP4C^{pp4\text{-}19C}$	16p11.2	57.63	5	86.84%
$SH2B1^{lnk}$	16p11.2 distal	57.47	6	84.21%
$CTNNB1^{arm}$	Core gene	54.60	7	81.58%
$TCTEX1D2^{CG5359}$	3q29	52.33	8	78.95%
$ATP2A1^{kum}$	16p11.2 distal	52.31	9	76.32%
$PIGZ^{CG3419}$	3q29	51.92	10	73.68%
$ZDHHC19^{app}$	3q29	49.77	11	71.05%
$MAPK3^{rl}$	16p11.2	49.52	12	68.42%
$SHANK3^{prosap}$	Core gene	48.99	13	65.79%
$CADPS2^{caps}$	Core gene	48.64	14	63.16%
$NCBP2^{Cbp20}$	3q29	47.23	15	60.53%
$CHD8^{kis}$	Core gene	46.41	16	57.89%
$SPNS1^{spin}$	16p11.2 distal	45.77	17	55.26%
$TAOK2^{dTao\text{-}1}$	16p11.2	42.44	18	52.63%
$DLG1^{dlg1}$	3q29	42.04	19	50.00%
$PTEN^{dpten}$	Core gene	40.51	20	47.37%
$CCDC101^{sgf29}$	16p11.2 distal	40.02	21	44.74%
$KIF22^{klp68D}$	16p11.2	38.48	22	42.11%
$TUFM^{efTuM}$	16p11.2 distal	38.40	23	39.47%
$BDH1^{CG8888}$	3q29	38.28	24	36.84%
$PAK2^{Pak}$	3q29	36.91	25	34.21%
$SCN1A^{para}$	Core gene	35.87	26	31.58%
$C16ORF53^{pa1}$	16p11.2	35.19	27	28.95%
$TBX6^{doc3\_2}$	16p11.2	33.25	28	26.32%
$OSTalpha^{CG6836}$	3q29	30.84	29	23.68%
$LRRC33^{CG7896}$	3q29	30.37	30	21.05%
$ALDOA^{ald}$	16p11.2	30.20	31	18.42%
$PCYT1A^{Cct1}$	3q29	28.48	32	15.79%
$ASPHD1^{asph}$	16p11.2	28.17	33	13.16%
$CORO1A^{coro}$	16p11.2	27.19	34	10.53%
$FBXO45^{Fsn}$	3q29	26.88	35	7.89%
$PIGX^{CG30381}$	3q29	26.37	36	5.26%
$YPEL3^{CG15309}$	16p11.2	25.28	37	2.63%
$CDIPT^{pis}$	16p11.2	23.76	38	0.00%
$FAM57B^{CG17841}$	16p11.2	NA	NA	NA

Average *Flynotyper* score, rank and percentile for one-hit *GMR-GAL4* knockdown of 16p11.2 homologs (shaded in grey) and other neurodevelopmental genes. *FAM57B*^{CG17841} knockdown caused lethality and was therefore not ranked.

Supplementary Table 3. Pathogenicity of 16p11.2 genes based on human allele frequency metrics

	CNV	pLI rank percentile	RVIS percentile
ALDOA	16p11.2 CNV	50.48	18.59
ASPHD1	16p11.2 CNV	42.04	28.93
C16orf54	16p11.2 CNV	25.51	
C16orf92	16p11.2 CNV	51.92	68.98
CDIPT	16p11.2 CNV	79.27	8.75
CORO1A	16p11.2 CNV	14.32	21.41
DOC2A	16p11.2 CNV	52.01	19.73
FAM57B	16p11.2 CNV	36.03	26.23
GDPD3	16p11.2 CNV	86.01	57.15
HIRIP3	16p11.2 CNV	51.91	79.52
INO80E	16p11.2 CNV	52.27	48.12
KCTD13	16p11.2 CNV	21.89	30.07
KIF22	16p11.2 CNV	79.74	34.88
MAPK3	16p11.2 CNV	32.46	26.23
MAZ	16p11.2 CNV	14.36	
MVP	16p11.2 CNV	56.27	12.05
PAGR1	16p11.2 CNV	29.88	
PPP4C	16p11.2 CNV	22.81	44.54
PRRT2	16p11.2 CNV	35.65	81.38
QPRT	16p11.2 CNV	27.57	80.73
SEZ6L2	16p11.2 CNV	41.52	6.58
SPN	16p11.2 CNV	43.63	34.60
TAOK2	16p11.2 CNV	6.13	0.60
TBX6	16p11.2 CNV	49.77	50.22
TMEM219	16p11.2 CNV	64.55	38.58
YPEL3	16p11.2 CNV	41.94	66.57
ELN	Williams syndrome	76.94	47.20
GTF2I	Williams syndrome	5.41	
KANSL1	17q21.31 syndrome	4.48	30.95
NSD1	Sotos syndrome	0.53	3.24
RAI1	Smith-Magenis syndrome	5.68	0.25
SHANK3	Phelan-McDermid syndrome	4.13	
TBX1	DiGeorge syndrome/VCFS	11.13	
UBE3A	Prader-Willi/Angelmans syndrome	4.93	19.54

Rank percentiles of ExAC loss-of-of function constraint metric (pLI) and intolerance to variation (RVIS) scores are shown for all 16p11.2 genes and causative genes for select syndromic CNVs. The pLI and RVIS rank percentile scores for several 16p11.2 genes, including *TAOK2*, *CORO1A*, *PPP4C*, *KCTD13*, *MAPK3*, and *MAZ*, are similar to known pathogenic and disease-associated genes, indicating that they all share an importance in human development.

SUPPLEMENTARY REFERENCES

1. Krishnan A, *et al.* Genome-wide prediction and functional characterization of the genetic basis of autism spectrum disorder. *Nat Neurosci* 19, 1454-1462 (2016).

2. Warde-Farley D, *et al.* The GeneMANIA prediction server: biological network integration for gene prioritization and predicting gene function. *Nucleic Acids Res* 38, W214-220 (2010).

3. Hu Y, *et al.* An integrative approach to ortholog prediction for disease-focused and other functional studies. *BMC Bioinformatics* 12, doi:10.1186/1471-2105-12-357 (2011).

4. Vilella AJ, Severin J, Ureta-Vidal A, Heng L, Durbin R, Birney E. EnsemblCompara GeneTrees: Complete, duplication-aware phylogenetic trees in vertebrates. *Genome Res* 19, 327-335 (2009).

5. Blake JA, Bult CJ, Kadin JA, Richardson JE, Eppig JT, Mouse Genome Database G. The Mouse Genome Database (MGD): premier model organism resource for mammalian genomics and genetics. *Nucleic Acids Res* 39, D842-848 (2011).

6. Jayachandran R, *et al.* Coronin 1 regulates cognition and behavior through modulation of cAMP/protein kinase A signaling. *PLoS Biol* 12, doi:10.1371/journal.pbio.1001820 (2014).

7. Blaker-Lee A, Gupta S, McCammon JM, De Rienzo G, Sive H. Zebrafish homologs of genes within 16p11.2, a genomic region associated with brain disorders, are active during brain development, and include two deletion dosage sensor genes. *Dis Model Mech* 5, 834-851 (2012).

8. Golzio C, *et al.* KCTD13 is a major driver of mirrored neuroanatomical phenotypes of the 16p11.2 copy number variant. *Nature* 485, 363-367 (2012).

9. McCammon JM, Blaker-Lee A, Chen X, Sive H. The 16p11. 2 homologs fam57ba and doc2a generate certain brain and body phenotypes. *Hum Mol Genet* 26, 3699-3712 (2017).

10. Chintapalli VR, Wang J, Dow JA. Using FlyAtlas to identify better Drosophila melanogaster models of human disease. *Nat Genet* 39, 715-720 (2007).

11. Celniker SE, *et al.* Unlocking the secrets of the genome. *Nature* 459, 927-930 (2009).

12. Wernet MF, Desplan C. Homothorax and Extradenticle alter the transcription factor network in Drosophila ommatidia at the dorsal rim of the retina. *Development* 141, 918-928 (2014).

13. Bharathi V, Pallavi SK, Bajpai R, Emerald BS, Shashidhara LS. Genetic characterization of the Drosophila homologue of coronin. *J Cell Sci* 117, 1911-1922 (2004).

14. Biggs WH, 3rd, *et al.* The Drosophila rolled locus encodes a MAP kinase required in the sevenless signal transduction pathway. *EMBO J* 13, 1628-1635 (1994).

15. Sarpal R, Ray K. Dynamic expression pattern of kinesin accessory protein in Drosophila. *J Biosci* 27, 479-487 (2002).
