# Supplementary material for: Pervasive genetic interactions modulate neurodevelopmental defects of the autism-associated 16p11.2 deletion in Drosophila melanogaster
Source: Nat Commun. 2018 Jun 29;9:2548. doi: 10.1038/s41467-018-04882-6 (PMC6026208; doi:10.1038/s41467-018-04882-6)
Supplement: Supplementary file 3 — Description of Additional Supplementary Files [file 41467_2018_4882_MOESM3_ESM.pdf]

Description of Additional Supplementary Files

File Name: Supplementary Data 1

Description: RNAi and deficiency fly stocks used for one-hit and two-hit experiments. Each tab of the Excel file shows fly and human genes, stock numbers, and sources for all lines, as well as the genotypes for deficiency lines.

File Name: Supplementary Data 2

Description: Quantitative PCR analysis of 16p11.2 homolog knockdowns. The first tab of this file lists the percentage of gene expression (mRNA) in 25 RNAi lines of 16p11.2 homologs with Elav-GAL4>Dicer2 driver at 25°C, or Elav-GAL4 without Dicer2 at room temperature for ALDOAald and PPP4Cpp4-19C RNAi lines (due to lethality with Dicer2). RNA levels were quantified using qPCR, normalized to rp49 and expressed relative to levels of RNA in control. The second tab lists the percentage of tiptop and numb expression (mRNA) in KK RNAi lines of 16p11.2 homologs. Although three genes displayed high levels of tiptop, we did not observe the distinct eye phenotypes documented with overexpression of tiptop in the tested KK lines and were able to rule out the effect of this overexpression on the eye phenotypes. All genes displayed normal levels of numb.

File Name: Supplementary Data 3

Description: Tested and validated pairwise knockdown models between 16p11.2 homologs. Tab 1 lists all tested pairwise interactions between 16p11.2 homologs, including the direction of interaction (enhancer or suppressor, highlighted in yellow), the method of testing (Flynotyper or manual scoring), the number of lines tested for each interaction, and the total number of interactions and interacting lines. Tab 2 lists manual scores for pairwise interactions; “ND” represents phenotypes not determined.

File Name: Supplementary Data 4

Description: Tested and validated pairwise knockdown models of 16p11.2 homologs with neurodevelopmental and transcriptome genes. This Excel file lists all tested pairwise interactions of 16p11.2 homologs with other neurodevelopmental genes (Tab 1) and genes identified from the transcriptome data (Tab 2), including the direction of interaction (enhancer or suppressor, highlighted in yellow), the method of testing (Flynotyper or manual scoring), the number of lines tested for each interaction, Gene Ontology annotations for cell proliferation or cell cycle, and the total number of interactions and interacting lines. Tab 3 lists manual scores for pairwise interactions at 30°C and 25°C; “ND” represents phenotypes not determined.

File Name: Supplementary Data 5

Description: Differentially-expressed genes in 16p11.2 knockdown flies. This file lists all differentially-expressed genes from RNA sequencing with log-fold change >1 or <-1 and false

discovery rate, and human homologs identified by DIOPT are included for each differentially-expressed gene.

File Name: Supplementary Data 6

Description: Input and connector genes identified in a human brain-specific interaction network. This file lists all genes connected within the human brain-specific gene interaction network. The input column identifies genes used as inputs into the network (16p11.2 genes, interacting neurodevelopmental genes, or genes identified from transcriptome data for KCTD13CG10465 and MAPK3rl knockdown). Also listed for each gene is the degree (number of edges connecting to each node), number of paths (number of shortest paths between genes that the node is part of), annotations for cell cycle or cell proliferation GO terms, and differential expression of that gene in the 16p11.2 knockdown models.

File Name: Supplementary Data 7

Description: Primers used in PCR experiments. Tab 1 of the Excel file shows sequences for the forward and reverse TAIL-PCR primers, while tab 2 lists all primers used in qPCR experiments.

File Name: Supplementary Data 8

Description: Statistical analysis of experimental data. This file shows all statistical information (test used, control and experimental data, test statistics, p-values, confidence intervals, and sample size) for data presented in the main and supplemental figures.
